# Metalloendopeptidase ADAM-like Decysin 1 (ADAMDEC1) in Colonic Subepithelial PDGFRα^+^ Cells Is a New Marker for Inflammatory Bowel Disease

**DOI:** 10.3390/ijms23095007

**Published:** 2022-04-30

**Authors:** Se Eun Ha, Brian G. Jorgensen, Lai Wei, Byungchang Jin, Min-Seob Kim, Sandra M. Poudrier, Rajan Singh, Allison Bartlett, Hannah Zogg, Sei Kim, Gain Baek, Masaaki Kurahashi, Moon-Young Lee, Yong-Sung Kim, Suck-Chei Choi, Kent C. Sasse, Samuel J. S. Rubin, Andres Gottfried-Blackmore, Laren Becker, Aida Habtezion, Kenton M. Sanders, Seungil Ro

**Affiliations:** 1 Department of Physiology and Cell Biology, University of Nevada School of Medicine, Reno, NV 89557, USA; seeunh@med.unr.edu (S.E.H.); brianjorgensen@med.unr.edu (B.G.J.); tobewl@gmail.com (L.W.); jin.jbc@gmail.com (B.J.); spoudrier@med.unr.edu (S.M.P.); rajans@med.unr.edu (R.S.); allisonbartlett@nevada.unr.edu (A.B.); hannahzogg@gmail.com (H.Z.); seik0918@gmail.com (S.K.); gain141@naver.com (G.B.); ksanders@med.unr.edu (K.M.S.); 2 Department of Physiology, Wonkwang Digestive Disease Research Institute and Institute of Wonkwang Medical Science, School of Medicine, Wonkwang University, Iksan 54538, Korea; 1@wku.ac.kr (M.-S.K.); lmy6774@hanmail.net (M.-Y.L.); wms89@hanmail.net (Y.-S.K.); 3 Department of Internal Medicine, Division of Gastroenterology and Hepatology, University of Iowa, Iowa City, IA 52242, USA; masaaki-kurahashi@uiowa.edu; 4 Department of gastroenterology, Wonkwang Digestive Disease Research Institute and Institute of Wonkwang Medical Science, School of Medicine, Wonkwang University, Iksan 54538, Korea; medcsc@wku.ac.kr; 5 Sasse Surgical Associates, Reno, NV 89502, USA; belrod@sassesurgical.com; 6 Gastroenterology and Hepatology, Stanford University School of Medicine, Stanford, CA 94305, USA; yrubin@stanford.edu (S.J.S.R.); andresg@stanford.edu (A.G.-B.); lsbecker@stanford.edu (L.B.); aidah@stanford.edu (A.H.)

**Keywords:** ADAMDEC1, mucosal PDGFRα^+^ cells, inflammatory bowel disease, Crohn’s disease, DSS colitis

## Abstract

Metalloendopeptidase ADAM-Like Decysin 1 (ADAMDEC1) is an anti-inflammatory peptidase that is almost exclusively expressed in the gastrointestinal (GI) tract. We have recently found abundant and selective expression of Adamdec1 in colonic mucosal PDGFRα^+^ cells. However, the cellular origin for this gene expression is controversial as it is also known to be expressed in intestinal macrophages. We found that Adamdec1 mRNAs were selectively expressed in colonic mucosal subepithelial PDGFRα^+^ cells. ADAMDEC1 protein was mainly released from PDGFRα^+^ cells and accumulated in the mucosal layer lamina propria space near the epithelial basement membrane. PDGFRα^+^ cells significantly overexpressed Adamdec1 mRNAs and protein in DSS-induced colitis mice. Adamdec1 was predominantly expressed in CD45^−^ PDGFRα^+^ cells in DSS-induced colitis mice, with only minimal expression in CD45^+^ CD64^+^ macrophages. Additionally, overexpression of both ADAMDEC1 mRNA and protein was consistently observed in PDGFRα^+^ cells, but not in CD64^+^ macrophages found in human colonic mucosal tissue affected by Crohn’s disease. In summary, PDGFRα^+^ cells selectively express ADAMDEC1, which is localized to the colon mucosa layer. ADAMDEC1 expression significantly increases in DSS-induced colitis affected mice and Crohn’s disease affected human tissue, suggesting that this gene can serve as a diagnostic and/or therapeutic target for intestinal inflammation and Crohn’s disease.

## 1. Introduction

Metalloendopeptidase ADAM-Like Decysin 1 (ADAMDEC1) was first identified as a unique member of the matrix metalloprotease (ADAM) family [1]. ADAMs are increasingly recognized as playing an important role in gut homeostasis and bowel inflammation [2]. ADAMDEC1 is abundantly, and almost exclusively, expressed in the gastrointestinal (GI) tract [3]. This GI-specific protein is associated with inflammatory bowel diseases including ulcerative colitis and Crohn’s disease [4,5], gastric adenocarcinoma [6], and colorectal cancer [7]. Although ADAMDEC1’s physiological roles are still largely unknown, a recent study has shown that this protein may have a protective effect against the development of Crohn’s disease [3]. However, studies of ADAMDEC1 in the GI tract have only been done on isolated macrophages or at tissue levels [3,4,5,8], and the gene expression profiles of other isolated cells within colonic mucosa have yet to be fully examined.

Previously, we obtained transcriptomes from various isolated GI cells including mucosal platelet-derived growth factor receptor-alpha-positive (PDGFRα^+^) cells, smooth muscle PDGFRα^+^ cells, interstitial cells of Cajal (ICC), and smooth muscle cells (SMC) isolated from mouse colon tissue [9,10,11]. Using the data obtained from these transcriptomes, we built the Smooth Muscle Transcriptome Browser that offers expression profiles, isoforms, and cDNA sequences, along with the open reading frames of individual genes expressed in each cell population as well as in whole gastrointestinal (GI) tissues [12]. Smooth Muscle Transcriptome Browser. Available online: https://med.unr.edu/physio/transcriptome (accessed on 23 January 2019) [12]. By analyzing these transcriptomes, we were able to identify that the *Adamdec1* gene is highly expressed in mucosal PDGFRα^+^ cells in a cell-specific manner [13]. However, the cell-specific expression of *Adamdec1* in mucosal PDGFRα^+^ cells is inconsistent with previous reports that found this gene to be expressed in human colonic macrophages [8]. 

In this study, in contrast to previous reports on macrophages, we found that *Adamdec1* is predominantly expressed in colonic mucosal PDGFRα^+^ cells in mice and humans, with little or no expression within macrophages. In addition, we found that the ADAMDEC1 protein is mainly localized near the intestinal epithelium and is greatly induced in murine tissue affected by colitis as well as human colonic mucosa tissue from Crohn’s disease-affected patients. These findings suggest an important role for colonic mucosal PDGFRα^+^ cells expressing ADAMDEC1 in the physiologic response to gut inflammation.

## 2. Results

### 2.1. Selective Expression of Adamdec1 within Intestinal PDGFRα^+^ Cells and Localization of the Protein to the Mucosal Lining

**Figure 1:**
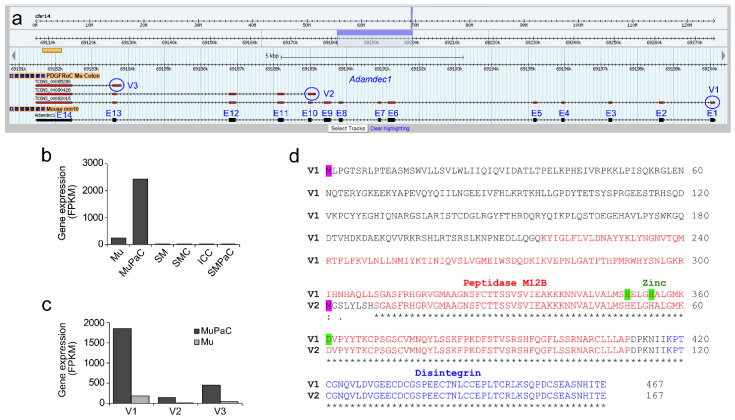
*Adamdec1* transcripts are selectively expressed in colonic mucosal PDGFRα^+^ cells. (**a**) A genomic map view of three *Adamdec1* transcriptional variants (V1-3) expressed in colonic mucosal PDGFRα^+^ cells. Three alternatively start exons (E1, E10 and E13) are circled (blue). (**b**) Expression levels of *Adamdec1* in isolated colonic cells and tissue: mucosa (Mu), mucosal PDGFRα^+^ cells (MuPaC), smooth muscle (SM), smooth muscle cells (SMC), interstitial cells of Cajal (ICC), and smooth muscle PDGFRα^+^ cells (SMPaC). (**c**) Expression levels of *Adamdec1* variants in Mu and MuPaC. (**d**) Alignment of predicted amino acid sequences of ADAMDEC1 variants. The open reading frame was identified for each variant and predicted amino acid sequences were aligned. Alternative starting methionines (M) are in pink. The peptidase M12B domain and Disintegrin domain are indicated by red and blue respectively. Three zinc binding sites are marked by green.

In our previous transcriptomic analysis of colonic smooth muscle and mucosal cells, we found that *Adamdec1* is selectively expressed in mucosal PDGFRα^+^ cells [13]. The *Adamdec1* gene contains 14 exons which are transcribed into three different transcriptional variants (V1, TCONS_00082015; V2, TCONS_00090428; V3, TCONS_00085286), alternatively starting at exons 1, 10, and 13 as shown on the Smooth Muscle Transcriptome Browser [12] (Figure 1A). The transcriptome data shows that Adamdec1 is highly expressed in mucosal PDGFRα^+^ cells (MuPaC), but with little to no expression in SMC, ICC, and smooth muscle PDGFRα^+^ cells (smPaC) (Figure 1B). This selective expression was consistent in whole mucosa (Mu) and smooth muscle (SM) tissue (Figure 1B). Among three Adamdec1 variants, V1 is the longest (2412 bp), then V2 (1579 bp), with V3 being the shortest (1213 bp). V1 is the dominant variant as it is the most highly expressed (Figure 1C). V1, V2, and V3 encode 467 aa, 167 aa, and 69 aa, respectively (Figure 1D). V1 contains the peptidase M12B domain containing three zinc-binding sites and a disintegrin domain, while V2 is truncated and only has half of the peptidase M12B domain and the full disintegrin domain at the C-terminus end. In contrast, the translated 69 aa sequence from V3 does not appear to have homology to any known protein, suggesting that V3 may not be translated. 

### 2.2. Reduction of Colonic Mucosal PDGFRα^+^ Cells in DSS-Induced Colitis

Expression of Adamdec1 is upregulated in DSS-induced colitis models [3]. Thus, *Pdgfrα^eGFP/+^* mice were treated with DSS treated water for eight days, after which the mice were allowed to recover by switching back to normal water for seven consecutive days (Supplementary Figure S1A). Administration of 2% DSS in drinking water for up to 8 days induced colitis in mice, as revealed by clinical symptoms (loss of body weight, diarrhea, rectal bleeding, and bloody stools) (Supplementary Figure S1B–E). DSS-induced colitis mice showed body weight loss at 8 days after beginning the DSS treatment (Supplementary Figure S1C). Rectal bleeding and bloody stools, both signs of colitis, were observed six days after DSS exposure (DSS 6D) and severe rectal bleeding was found eight days after DSS treatment (DSS 8D). Rectal bleeding was no longer found seven days after the DSS treated mice were switched to normal water (Supplementary Figure S1B–E). Blood was also found in the colonic mucosa at DSS 6D and 8D but was not found in control mice or in mice who were allowed seven days of recovery after being switched to normal water from DSS treated water (Rec 7D). Colonic crypt length on DSS-induced colitis mice was considerably shortened compared with control mice (Supplementary Figure S1F–H). Inflammatory cytokines were also increased due to DSS-induced colitis. We tested expression of interleukin-6 (IL-6), a well-established indicator of inflammation, in blood and colonic tissue using ELISA and found that IL-6 was increased in DSS-induced colitis mice and decreased after switching back to normal water (Rec 7D) (Supplementary Figure S1I).

**Figure 2:**
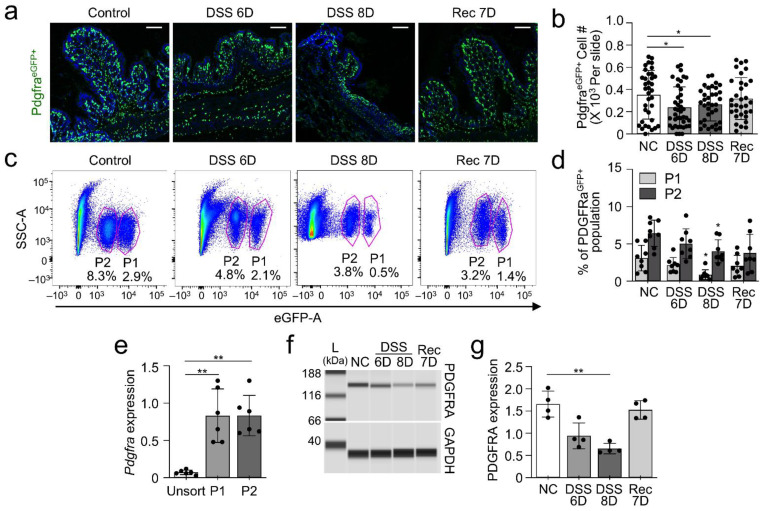
Loss of mucosal PDGFRα^+^ cells in DSS-induced colitis mouse model. PDGFRα^+^ cells in colonic mucosa identified by eGFP. (**a**) Reduction of PDGFRα^+^ cells in DSS-treated mice after 6 days (DSS 6D)- and 8 days (DSS 8D) compared to non-treated control (NC) mice and recovery mice for 7 days post DSS treatment (Rec 7D). DSS-induced proximal and distal colonic mucosa and restoration of the cells in recovery colon. (**b**) Quantification of the number of PDGFRα^+^ cells in (**a**) (*n* = 20). * *p* < 0.05, versus NC groups. (**c**) Changes of PDGFRα^+^ cell populations P1 and P2 in DSS-treated mice, compared with control and recovered mice. (**d**) Quantification of PDGFRα^+^ cell populations P1 and P2 in (c) (*n* = 10). * *p* < 0.05, versus NC groups (**e**) *Pdgfra* mRNA expression in PDGFRα^+^ cell populations P1 and P2 (*n* = 5). ** *p* < 0.01, versus unsorted. (**f**) Decrease of PDGFRA protein expression in colonic mucosa tissue from DSS-induced colitis mouse model. (**g**) Quantification of PDGFRA in (**f**) normalized by GAPDH (*n* = 4). ** *p* < 0.01, versus NC groups.

Next, we wanted to know if there would be a change in the quantity of enhanced green fluorescent protein (eGFP^+^) PDGFRα^+^ cells or other physiological changes in DSS-induced colitis tissue from *Pdgfrα^eGFP/+^* mice. In DSS 6D and 8D, we observed a loss of *Pdgfra-eGFP*^+^ cells and damage to mucosal epithelium of the colon (Figure 2A,B). In contrast, no loss of *Pdgfra-eGFP^+^* cells were found in the control or Rec 7D mice (Figure 2A–D). Additionally, less *Pdgfra-eGFP*^+^ cells were found in sorted cells from the DSS treated colon mucosa as compared to control mice (Figure 2B,D). Previously reported data showed two distinct populations of *Pdgfra-eGFP^+^* cells in murine colonic mucosa: cells with brighter eGFP fluorescence (bright eGFP, P1) and cells with dimmer eGFP fluorescence (dim eGFP, P2) [14]. Both P1 and P2 populations had specific expression of *Pdgfra* mRNA (Figure 2E). Both P1 and P2 populations of *Pdgfra-eGFP^+^* cells were reduced in DSS 6D and 8D mice, compared to control mice, and P1 cell numbers were slightly restored in Rec 7D mice (Figure 2B,D). Additionally, PDGFRA protein expression was decreased in all DSS-induced colitis mice, and increased in Rec 7D (Figure 2F,G). 

### 2.3. Upregulation of Adamdec1 Transcripts and Protein in Colonic Mucosal PDGFRα^+^ Cells in DSS-Induced Colitis 

**Figure 3:**
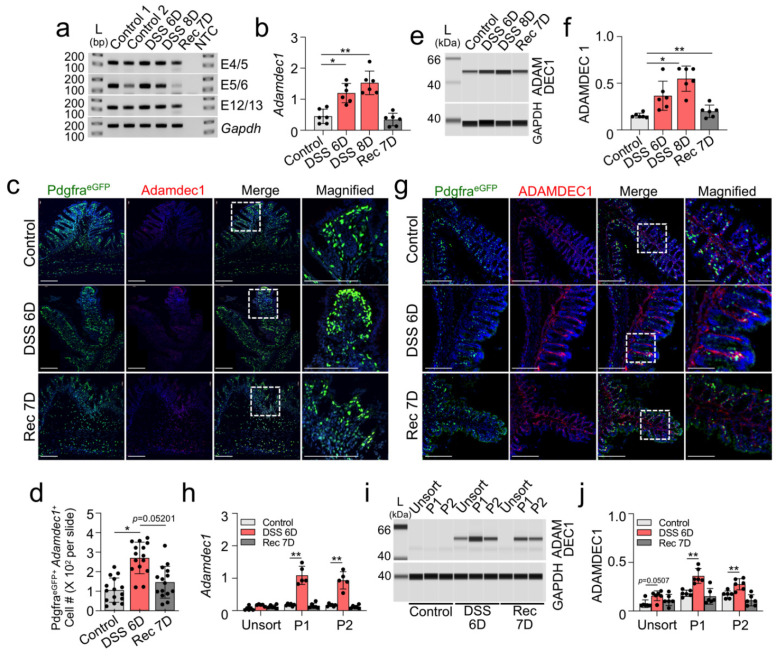
Induction of ADAMDEC1 in PDGFRα^+^ cells in DSS-induced colitis. (**a**) Expression of *Adamdec1* in DSS-induced colonic mucosa at 6 and 8 days post DSS treatment (DSS 6D and 8D) and recovery mucosa at 7 days (Rec 7D: switched to water after DSS treatment) measured by RT-PCR. Three independent primer sets spanning exons E4/5, E5/6, and E12/13 of *Adamdec1* were used. (**b**) Quantitative analysis of *Adamdec1* mRNA expression in DSS-induced colonic mucosa and recovery mucosa (*n* = 5–7) measured by qPCR. *Gapdh* was used as an endogenous control. (**c**) RNA in situ hybridization showing increased expression of *Adamdec1* in PDGFRα^+^ cells (*Pdgfra-eGFP^+^* cells) in DSS-induced colitis. Scale bars are 100 μm. The dotted white box in the “Merge” image indicates the selected area of zoom for the “Magnified” image of the figure. (**d**) Quantification of number of *Pdgfra-eGFP*^+^*Adamdec1* mRNA^+^ cells in (c) (*n* = 5). (**e**) Changes of ADAMDEC1 proteins in DSS-induced mucosa and recovery mucosa. GAPDH was used as a loading control. (**f**) Quantification of ADAMDEC1 in e normalized by GAPDH (*n* = 6). (**g**) Increased accumulation of ADAMDEC1 in the epithelial layers of DSS-induced colonic mucosa. Scale bars are 100 μm. The dotted white box in the “Merge” image indicates the selected area of zoom for the “Magnified” image of the figure. (**h**) Quantitative analysis of *Adamdec1* mRNA expression in P1 and P2 of PDGFRα^+^ cells isolated from DSS-induced colonic mucosa and recovery mucosa along with unsorted cells measured by qPCR (*n* = 5–6). (**i**) ADAMDEC1 protein expression in P1 and P2 of PDGFRα^+^ cells isolated from DSS-induced colonic mucosa and recovery mucosa along with unsorted cells. (**j**) Quantification of ADAMDEC1 in (i) normalized by GAPDH (*n* = 6). * *p* < 0.05 and ** *p* < 0.01, versus control groups.

Next, we examined expression changes of Adamdec1 in the DSS-induced colitis mouse model. Various Adamdec1 transcript expression levels were measured in isolated mucosa tissue with three different exon specific primer pairs by qPCR and all primer pairs showed significantly increased Adamdec1 levels in DSS 6D and 8D mice, which returned to control levels in Rec 7D mice (Figure 3A,B). In addition, RNA in situ hybridization confirmed increased expression of Adamdec1 mRNAs in PDGFRα^+^ cells at DSS 6D (Figure 3C,D). ADAMDEC1 protein expression gradually increased at 6D and 8D while PDGFRA decreased (Figure 3E,F). The increased ADAMDEC1 protein was found at the epithelial line and around PDGFRα^+^ cells in colon mucosa at DSS 6D (Figure 3G). The increased mRNA and protein expression was also found in P1 and P2 of MuPαCs isolated from DSS 6D colon mucosa, with expression returning to levels close to control in Rec 7D mice (Figure 3H–J).

**Figure 4:**
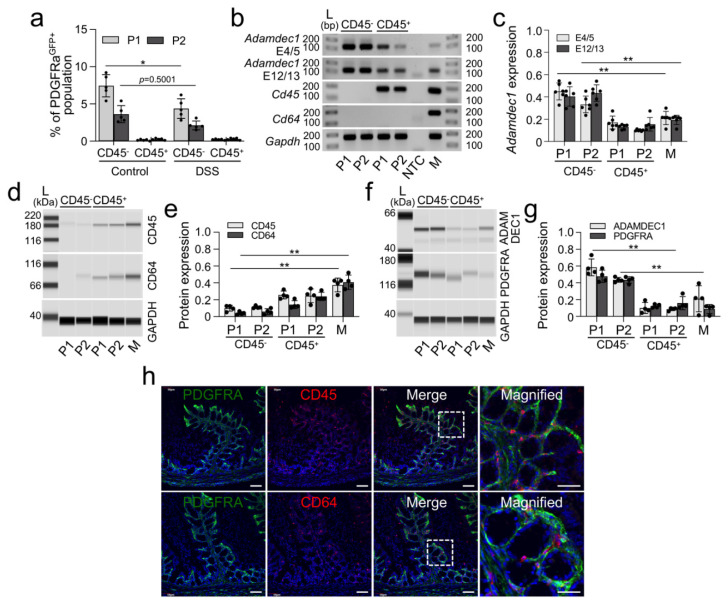
Predominant expression of *Adamdec1* in mucosal CD45^−^ PDGFRα^+^ cells. (**a**) PDGFRα^+^ (*Pdgfra-eGFP^+^*) populations (P1 and P2) in CD45^−^ and CD45^+^ cells identified by flow cytometry at 6 days post DSS treatment (DSS 6D) and control colonic mucosa. Percentage of PDGFRα^+^ cell populations P1 and P2 in DSS 6D and control (*n* = 5). *p < 0.05, versus control groups (**b**) Expression of *Adamdec1* in isolated P1 and P2 of colonic mucosal CD45^−^ and CD45^+^ cells and CD45^+^CD64^+^CD14^+^ (macrophages; M) cells at 6 days post DSS treatment colonic mucosa, examined by RT-PCR with primer sets for *Adamdec1, Cd45 and Cd64*. *Gapdh* was used as a loading control. (**c**) Quantitative analysis of *Adamdec1* expression in PαC (P1 and P2) of CD45^−^ cells and CD45^+^ cells (*n* = 5) isolated from DSS-induced colitis colonic mucosa measured by qPCR. (**d**) Expression of CD45 and CD64 protein expression in isolated P1 and P2 of colonic mucosal CD45^−^ and CD45^+^ cells and M cells isolated from DSS-induced colitis colonic mucosa. (**e**) Quantification of CD45 and CD64 in (**d**) normalized by GAPDH (*n* = 4). (**f**) Expression of ADAMDEC1 protein expression in isolated P1 and P2 of colonic mucosal CD45^−^ and CD45^+^ cells and M cells from DSS-induced colitis colonic mucosa. (**g**) Quantification of ADAMDEC1 in (**f**) normalized by GAPDH (*n* = 4). (**h**) Most PDGFRα^+^ cells do not overlap with CD45^+^ or CD64^+^ cells in DSS 6D colonic mucosa. Scale bars are 50 μm. The dotted white box in the “Merge” image indicates the selected area of zoom for the “Magnified” image of the figure. * *p* < 0.05 and ** *p* < 0.01.

Adamdec1 expression has also been identified in intestinal macrophages [8]. Thus, we measured expression of Adamdec1 mRNA and protein in control and DSS-induced colonic mucosa. Macrophages were defined as live CD45^+^CD64^+^CD14^+^ cells [15,16,17,18]. PαCs (P1 and P2) of CD45^−^ cells, CD45^+^ cells, and CD45^+^CD64^+^CD14^+^ macrophage cells were isolated respectively by flow cytometry (Figure 4A and Supplementary Figure S2). ADAMDEC1 mRNA and protein expression was confirmed to be greatest in P1 CD45^−^ followed by P2 CD45^−^, P1 CD45^+^, P2 CD45^+^, and CD45^+^CD64^+^CD14^+^ macrophages (Figure 4A–G). Additionally, immunofluorescence analysis confirmed that PDGFRα^+^ cells in both control and DSS-induced colonic mucosa did not colocalize with CD45^+^ and CD64^+^ cells (Figure 4H). PDGFRA and CD45 or CD64 did not colocalize with their respective genetic signatures and did not overlap in analysis. These data suggest that Adamdec1 is predominantly expressed in resident PDGFRα^+^ cells in the mucosa while being expressed very minimally in macrophages. 

### 2.4. ADAMDEC1 Is Upregulated in Human Tissue Affected by IBD

**Figure 5:**
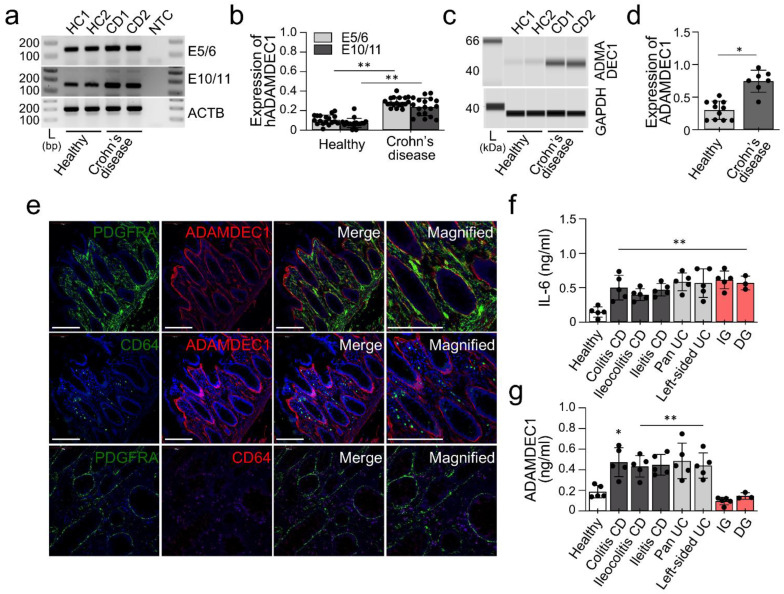
Induction of ADAMDEC1 in human IBD samples. (**a**) Expression of *ADAMDEC1* in Crohn’s disease (CD)-affected colon and healthy controls (HC) examined with two primer sets (E5/6 and E10/11). NTC, non-template control; L, DNA marker. *ACTB* was used as an endogenous control. (**b**) Quantitative analysis of *ADAMDEC1* in CD (*n* = 15) and HC (*n* = 10). *ACTB* was used for an endogenous control. (**c**) Changes of ADAMDEC1 proteins in Crohn’s disease-affected colon. GAPDH were used as a loading control. (**d**) Quantification of ADAMDEC1 in (**c**) normalized by GAPDH (*n* = 7–10). (**e**) Localization of ADAMDEC1 protein in colonic PDGFRα^+^ cells (PDGFRA) and surrounding epithelial layers, but not in CD64^+^ cells. Scale bars are 50 μm. (**f**) Expression of IL-6 protein expression in plasma samples from CD colitis, CD ileocolitis, CD ileitis, pan ulcerative colitis (UC), left-sided UC, idiopathic gastroparesis (IG), and diabetic gastroparesis (DG) by ELISA (*n* = 3–5 per groups). (**g**) Expression of ADAMDEC1 protein expression plasma samples from CD colitis, CD ileocolitis, CD ileitis, pan UC, left-sided UC, IG and DG by ELISA (*n* = 3–5 per groups). * *p* < 0.05 and ** *p* < 0.01, versus HC.

Lastly, we examined if the upregulation of Adamdec1 observed in our DSS mouse colitis model is consistent in human tissue affected by Crohn’s disease. Using two different exon-spanning primer sets in RT-PCR, we found that while both healthy and Crohn’s disease affected colonic tissues expressed *ADAMDEC1* (Figure 5A,B). Expression of *ADAMDEC1* was significantly increased in Crohn’s disease-affected colonic tissue compared to healthy colonic tissue (Figure 5B). ADAMDEC1 protein was detected (Figure 5C) and was found at notably higher levels in Crohn’s disease-affected colonic tissue (Figure 5D). Additionally, ADAMDEC1 was detected through immunohistochemistry within PDGFRα^+^ cells in Crohn’s disease-affected colonic mucosa (Figure 5E). ADAMDEC1 signals were detected within PDGFRα^+^ cells, however, PDGFRα^+^ cells did not colocalize with CD64 nor did their respective genetic signatures overlap in analysis (Figure 5E). These results indicate that human *ADAMDEC1* is specifically expressed in human PDGFRα^+^ cells, not CD64^+^ cells. This increase of both mRNA and protein of ADAMDEC1 in Crohn’s disease-affected colonic mucosa is consistent with our DSS-induced colitis mouse model (Figure 3). These data suggest that *ADAMDEC1* is overexpressed in PDGFRα^+^ cells in Crohn’s disease-affected colonic mucosa and expressed at a minimal level in macrophages.

Next, we analyzed the circulating expression levels of IL-6 inflammatory cytokines and ADAMDEC1 in plasma samples from various IBD subtypes. We also compared samples from gastroparesis patients, who have mucosal immune dysregulation of the stomach [19], but no known IBD. We confirmed the expression of IL-6 and ADAMDEC1 in plasma samples from colitis, ileocolitis, ileitis, polyarteritis nodosa (Pan), ulcerative colitis (left-sided), idiopathic gastroparesis (IG) and diabetic gastroparesis (DG) by ELISA (Figure 5F,G). IL-6 was found to be increased in all diseases (Figure 5F) but ADAMDEC1 was only significantly increased in all IBD subtypes and not gastroparesis (Figure 5G). These results suggest that the expression of ADAMDEC1 is specifically influenced by IBD, and is consistent with previous mouse data [3].

## 3. Discussion

In this study, we discovered that *Adamdec1* is mainly transcribed in colonic mucosal PDGFRα^+^ cells in both mice and humans. The mRNA and protein expression of the *Adamdec1* gene was induced in both DSS-induced colitis mouse models and human Crohn’s disease patients. These findings show insight into a potential new role of colonic mucosal PDGFRα^+^ cells as antagonists of inflammation within the GI tract. 

ADAMDEC1, also known as decysin, is most abundantly expressed in the middle and lower GI tract and with little expression being found in other tissues including lymph nodes, the thymus and spleen [1,3,8]. The ADAMDEC1 protein is most highly expressed in proximal and distal colonic mucosa and is moderately expressed in duodenal, jejunal, and ileal mucosa [3]. However, since other ADAM family members (ADAM8 [20], ADAM17 [21], ADAM28 [22], and MADAM [23]) are commonly expressed in immune cells, ADAMDEC1 was initially searched for in immune and blood cells. Previous studies report that ADAMDEC1 transcripts were found in macrophages, activated dendritic cells, in human tonsils [1] and mouse spleen [24], and recently in platelets [25]. In addition, *Adamdec1* transcripts have been found in macrophages isolated from colonic mucosa biopsy specimens [8]. 

In contrast, we found *Adamdec1* transcripts were enriched in the transcriptome of colonic mucosal PDGFRα^+^ cells and selectively expressed in these cells over colonic smooth muscle PDGFRα^+^ cells [10], SMC [12], and ICC [11]. PDGFRα^+^ cells, SMC, and ICC within the smooth muscle are believed to originate from the same mesenchymal precursor cells [26,27]. PDGFRα^+^ cells develop into SMC and ICC during embryonic development [26] and SMC can dedifferentiate into PDGFRα^+^ cells when smooth muscle is induced into hypertrophy [28]. Colonic mucosal PDGFRα^+^ cells were expressed in the subepithelial myofibroblasts and in the mesenchymal cells and play an important functional role in villus morphogenesis and regeneration. Our previously study shows that Bmp7, Bmp5, Wnt5a, Wnt4, and Wif1 were more highly specific for mucosal PDGFRα^+^ cells [13]. *Adamdec1* appears to be selectively expressed in mucosal PDGFRα^+^ cells, suggesting this gene is one of the best markers for these PDGFRα^+^ cells. 

PDGFRα^+^ cells are not derived from hematopoietic stem cells (CD45) in the peripheral blood and bone marrow, which give rise to macrophages and dendritic cells. We showed that mature PDGFRα^+^ cells are CD45^−^ cells (Figure 4). *Adamdec1* is highly expressed in CD45^−^ cells in mice that include PDGFRα^+^ cells, but minimally expressed in CD45^+^ cells. Our mouse data is consistent with our human colon mucosa immunofluorescence analysis which found the ADAMDEC1 protein mainly localizes to the epithelial barrier but does show some colocalization with PDGFRα^+^ cells, which was not found in macrophages (Figure 3 and Figure 5). Since *Adamdec1* mRNA, not protein, was detected in macrophages [3,29], it is possible that the transcripts may have been obtained from PDGFRα^+^ cells phagocytosed by macrophages. Furthermore, we were able to find that ADAMDEC1 expression in PDGFRα^+^ cells is increased by colonic inflammation. *Adamdec1* has been shown to be upregulated in a DSS-induced colitis mouse model [3]. We were able to confirm this upregulation of *Adamdec1* mRNA and protein in PDGFRα^+^ cells in DSS-induced mouse models (Figure 3 and Figure 4). Furthermore, the removal of the *Adamdec1* gene in mice induces a severe inflammatory response and bacteremia, which increases mortality, suggesting a protective role of ADAMDEC1 against bacterial insults into mucosa [3]. Additionally, *Adamdec1* knockout mice showed increased sensitivity to the DSS colitis, associated with neutrophilic (CD1B^+^ GR1^+^ F480^−^) infiltrate [3]. However, *ADAMDEC1* has reduced levels of expression in macrophages in Crohn’s disease patients [5]. Conversely, our data shows that expression of both *ADAMDEC1* mRNA and protein is indeed upregulated in Crohn’s disease patients (Figure 5). Our data suggest that study of the expression of ADAMDEC1 should be focused on PDGFRα^+^ cells in Crohn’s disease patients, and not in macrophages. Induction of the protein in Crohn’s disease aligns with the results of our DSS-induced colitis mouse model. Our data combined with the results of the transgenic *Adamdec1* knockout phenotype indicate that induction of *Adamdec1* may be a secondary defensive response to inflammation and that increased ADAMDEC1 contributes to loss of PDGFRα+ cells in DSS-induced colitis. 

It is largely unknown how ADAMDEC1 plays a protective role in Crohn’s disease. ADAMDEC1 is a new member of the ADAM gene superfamily that functions as adhesion proteins and/or endopeptidases [30]. ADAMDEC1 is known to be made as a precursor (~53 kDa), which may be cleaved and processed into a mature protein with a molecular weight of ~33 kDa [31] and ectopically expressed in HEK 293 cells [32]. However, we found only one band of the full-length protein and could not find the cleaved protein in vivo in mice or humans (Figure 2 and Figure 5). The protein is secreted as an active metalloprotease, which cleaves macromolecular substrates such as α2-macroglobulin and casein [32] as well as epidermal growth factor precursor on platelet membrane [25]. Our data from mice and humans showed *Adamdec1* is expressed in the subepithelial PDGFRα^+^ cells, but the protein is mainly localized within the mucosa layer closer to the lumen (Figure 2 and Figure 5). Further studies should investigate how ADAMDEC1 is transported to the epithelial basement membrane from PDGFRα^+^ cells and what the protein targets are within the layer. 

In summary, we identified ADAMDEC1 as a novel marker for intestinal inflammation and Crohn’s disease and it is selectively expressed in colonic mucosal PDGFRα^+^ cells. This identification opens a new door into a potentially vital role of PDGFRα^+^ cells in intestinal inflammatory diseases. 

## 4. Methods and Materials

### 4.1. Animal and Tissue Preparation 

*Pdgfr*α*^eGFP/+^* mice were obtained from Jackson Laboratory [33]. Colonic mucosa was isolated and then trypsinized to collect PDGFRα^+^ cells using flow cytometry. The animal protocol was approved by the Institutional Animal Care and Use Committee at the University of Nevada-Reno Animal Resources. 

### 4.2. Dextran Sulfate Sodium Treatment

Colitis was induced by oral administration of 2% dextran sulfate sodium (Dextran Sulfate Sodium Salt, 36,000–50,000 M.Wt., Colitis Grade) (MP Biomedicals, Irvine, CA, USA) in drinking water for either 6 or 8 days. Control mice received drinking water only. Our recovery mice had DSS solution replaced with normal drinking water for 7 days following the prescribed DSS treatment regimen.

### 4.3. Human Samples

Frozen colonic tissue and plasma samples were obtained from patients affected with Crohn’s disease (both diseased and non-diseased tissues). These human samples were obtained through approved IRB protocols from Stanford University School of Medicine, Sasse Surgical Associates, Wonkwang University School of Medicine, and University of Nevada, Reno. Plasma was obtained from the tube supernatants after 10 min of centrifugation (800 g) at room temperature, aliquoted, and frozen at −80 °C until further use. Clinical characteristics of the gastroparesis samples were previously reported [34]. All clinical characteristics of human samples used in this study can be found in Supplementary Table S2.

### 4.4. Fluorescence-Activated Cell Sorting (FACS)

Cells were dispersed from the colonic mucosa and green fluorescent protein (GFP)^+^ PDGFRα^+^ cells were sorted from dispersed cells using FACS as previously described [10]. Isolated PαC were lysed and pooled from approximately 30 mice (15 males and 15 females), which were used as one collective sample in the isolation of total RNAs. Macrophage cells were incubated with APC-Cy7 Anti-CD45, PE anti-mouse CD64, and PE-Cy7 anti-mouse CD14 followed by washing with PBS/1% FBS. Resuspended cells had Hoechst 33258 (1 μg/mL; Biotium, Fremont, CA, USA) added as a viability marker. Cells were sorted and analyzed using the BD FACSAria II (BD Biosciences, San Jose, CA, USA) Special Order Research Product with a 130 μm nozzle with sheath pressure at 12 psi. The 355 nm laser excited Hoechst 33258 with a 450/50 nm bandpass filter. The GFP^+^ PDGFRα^+^ cells were excited using a 488 nm laser with a 530/30 nm bandpass filter. A neutral density filter was used on the forward scatter detector due to the high forward scatter properties. Cells that were GFP^+^ PαC CD45^−^ CD64^−^ CD14^−^ or GFP^−^ CD45^+^ CD64^+^ CD14^+^ were sorted into PBS/1% FBS. Acquisition was performed on BD FACSDiva 8.0 and TreeStar Flowjo (Ashland, OR, USA) was used to generate figures.

### 4.5. Isolation of Total RNAs

Total RNA was isolated from the colonic mucosa of both mice and human samples using the mirVana miRNA isolation kit (Life Technologies, Carlsbad, CA, USA), according to the manufacturer’s specification. The quality of total RNAs was analyzed using a NanoDrop 2000 Spectrometer (Thermo Scientific, Waltham, MA, USA) and a 2100 Bioanalyzer (Agilent Technologies, Santa Clara, CA, USA).

### 4.6. Real Time PCR

We used 2 ug of extracted total RNA in a 40 uL reverse transcription reaction using SuperScript III Reverse Transcriptase (Invitrogen, Waltham, MA, USA), after DNA removal with DNA-free DNA Removal Kit (Ambion, Austin, TX, USA). cDNA libraries were constructed through reverse transcription of the mRNA isolated from FACS-purified PDGFRα^+^ cells as previously described [10,13]. Reverse-transcription polymerase chain reaction (RT-PCR) and quantitative PCR (qPCR) analyses of cDNA were performed as previously described [10]. qPCR was carried out on a (7900HT) Fast Real-Time PCR System (Applied Biosystems, Waltham, MA, USA) utilizing SYBR Green. The expression levels of *Adamdec1*, *Cd45* and *Cd64* mRNA were normalized to *Gapdh* expression. All primer sequences used can be found in Supplementary Table S3.

### 4.7. Confocal Microscopy and Immunofluorescence Analysis

Frozen human and murine colonic tissues were cut onto vectabonded slides through cryostat sectioning. Tissue was then visualized through confocal microscopy to capture immunofluorescence staining or endogenous GFP fluorescence as previously described [10]. Primary antibodies against the following antigens were used in immunofluorescence staining: anti-ADAMDEC1 anti-PDGFRA, and anti-CD64. All antibodies used can be found in Supplementary Table S4. Images were collected using Fluoview (FV10-ASW 3.1) Viewer software (Olympus, Shinjuku City, Tokyo, Japan) with an Olympus FV1000 confocal laser scanning microscope.

### 4.8. Automated Immunoblot

Protein was extracted from the colon tissue, isolated colonic mucosa tissue samples of *Pdgfr*α^eGFP/+^ mice and human GI tissues by grinding with a mortar and pestle in modified RIPA buffer (Thermofisher, Waltham, MA, USA). Automated immunoblot was performed using WES (ProteinSimple, San Jose, CA, USA) on human and mouse protein samples. Quantification of banding patterns were performed using Compass software (v4.0.0) (ProteinSimple, San Jose, CA, USA) for WES. All antibodies used can be found in Supplementary Table S4. 

### 4.9. RNA In Situ Hybridization

The RNA was labeled with biotin following the reactions according to the instructions of the manufacturer (Roche, Basel, Switzerland). Following hybridization with Biotinylated probes, samples were incubated with biotinylated-Ubb or biotinylated-Adamdec1. After incubation, samples were labeled with streptavidin-594 (Jackson ImmunoResearch, West Grove, PA, USA) and the slides were counterstained with DAPI in a mounting solution (Abcam, Cambridge, UK). Images were collected using Fluoview (FV10-ASW 3.1 Viewer software; Olympus, Shinjuku City, Tokyo, Japan) with an Olympus FV1000 (Olympus, Shinjuku City, Tokyo, Japan) confocal laser scanning microscope.

### 4.10. Enzyme-Linked Immunosorbent Assay (ELISA)

IL-6 and ADAMDEC1 measurements were gathered from both mouse tissue and serum and human plasma. Tissue protein (for detected ELISA kit) was isolated using Trizol LS (Thermofisher, Waltham, MA, USA), according to the manufacturer’s specifications. ELISA were performed using mouse IL-6 Kit (Invitrogen, Waltham, MA, USA), human IL-6 Kit (Invitrogen, Waltham, MA, USA), and human AMDADEC1 ELISA kit (MyBioSource, San Diego, CA, USA), according to the manufacturers’ specification

### 4.11. Statistical Analysis

All the data obtained in the present study were compared using the unpaired student’s *t*-test to determine whether any differences were statistically significantly different when *p* ≤ 0.05 (*) or *p* ≤ 0.01 (**). The means of normalized values from at least three independent experiments were determined and subjected to student’s *t*-test.

## Data Availability

The RNA-seq data are able at the NCBI GEO with the following accession numbers: Mu Colon (Colon mucosa), GSM1388414 and PDGFRαC Mu Colon (PDGFRα cells in colonic mucosa), GSM1388415.

## Supplementary Materials

The following supporting information can be downloaded at: https://www.mdpi.com/article/10.3390/ijms23095007/s1.
