# Supplementary material for: Metalloendopeptidase ADAM-like Decysin 1 (ADAMDEC1) in Colonic Subepithelial PDGFRα+ Cells Is a New Marker for Inflammatory Bowel Disease
Source: Int J Mol Sci. 2022 Apr 30;23(9):5007. doi: 10.3390/ijms23095007 (PMC9103908; doi:10.3390/ijms23095007)
Supplement: Supplementary file 1 [file ijms-23-05007-s001.zip › Suplementary Figure and Tables legends.pdf]

Supplementary Figure S1. Pathological development of colitis in DSS-induced mice. **(a)** A study design of DSS-induced colitis and recovery mice. **(b)** DSS-treated mice after 6 days (DSS 6D) and 8 days (DSS 8D) showing rectal bleeding in a dotted circle compared to non-treated control mice (NC) and recovery mice for 7 days post DSS treatment (Rec 7D). Colon with cecum was dissected from each group to confirm rectal bleeding (arrows). **(c)** Body weight changes. **(d, e)** Stool and bleeding scores in DSS 6D, 8D, and Rec 7D mice, compared to NC. **(f)** Length of colon in DSS 6D, 8D, and Rec 7D mice, compared to NC. **(g)** H&E staining of colon. **(h)** Mucosal fold length of colon. **(i)** Expression of IL-6 in whole blood and colon in DSS 6D, DSS 8D and Rec 7D compared to NC by ELISA. (n=7-10). *$p < 0.05$ and **$p < 0.01$, versus NC.

Supplementary Figure S2. PDGFRα^+ cells identified by flow cytometry in DSS induced colitis mice. **(a)** PDGFRα^+ (*Pdgfra-eGFP*$^+$) populations (P1 and P2) in CD45$^-$ and CD45$^+$ cells identified by flow cytometry at 6 days post DSS treatment (DSS 6D) and control colonic mucosa. **(b)** Macrophage (CD14$^+$64$^+$) population in CD45$^+$ and CD45$^-$ cells identified by flow cytometry at DSS 6D.

Supplementary Table S1. Assessment of disease activity index (DAI)

Supplementary Table S2. Clinical information of human samples used in this study

Supplementary Table S3. Oligonucleotides used in this study

Supplementary Table S4. Antibodies used in this study
