# Supplementary figures and images for: Metalloendopeptidase ADAM-like Decysin 1 (ADAMDEC1) in Colonic Subepithelial PDGFRα+ Cells Is a New Marker for Inflammatory Bowel Disease
Source: Int J Mol Sci. 2022 Apr 30;23(9):5007. doi: 10.3390/ijms23095007 (PMC9103908; doi:10.3390/ijms23095007)

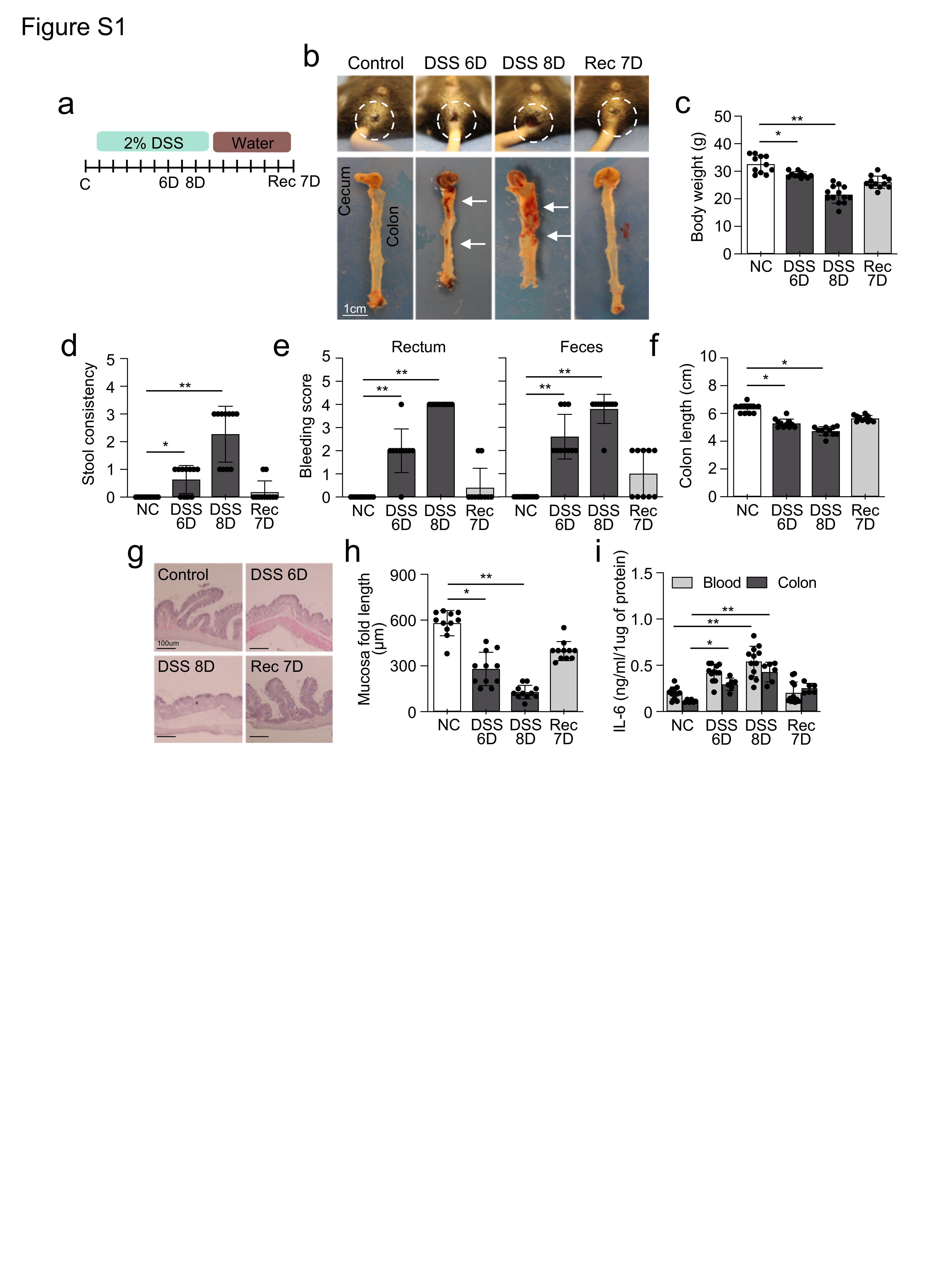

Supplement: Supplementary file 1 [file ijms-23-05007-s001.zip › Supplementary Figure S1.tif]

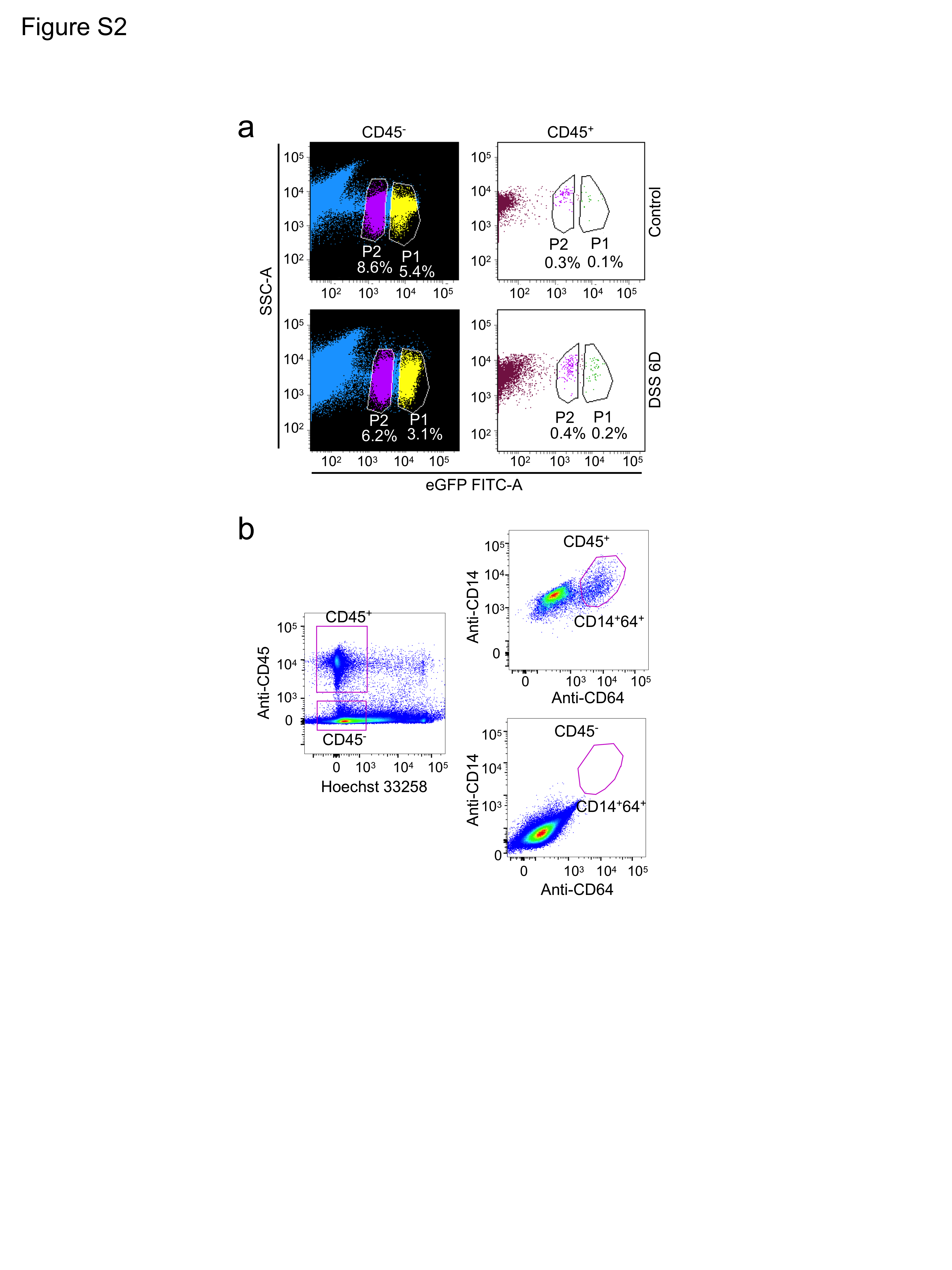

Supplement: Supplementary file 1 [file ijms-23-05007-s001.zip › Supplementary Figure S2.tif]
